# Investigation of the electrical conductivity of propylene glycol-based ZnO nanofluids

**DOI:** 10.1186/1556-276X-6-346

**Published:** 2011-04-19

**Authors:** Steven Bryan White, Albert Jau-Min Shih, Kevin Patrick Pipe

**Affiliations:** 1 Department of Mechanical Engineering, University of Michigan, Ann Arbor, MI 48109-2125, USA

## Abstract

Electrical conductivity is an important property for technological applications of nanofluids that has not been widely studied. Conventional descriptions such as the Maxwell model do not account for surface charge effects that play an important role in electrical conductivity, particularly at higher nanoparticle volume fractions. Here, we perform electrical characterizations of propylene glycol-based ZnO nanofluids with volume fractions as high as 7%, measuring up to a 100-fold increase in electrical conductivity over the base fluid. We observe a large increase in electrical conductivity with increasing volume fraction and decreasing particle size as well as a leveling off of the increase at high volume fractions. These experimental trends are shown to be consistent with an electrical conductivity model previously developed for colloidal suspensions in salt-free media. In particular, the leveling off of electrical conductivity at high volume fractions, which we attribute to counter-ion condensation, represents a significant departure from the "linear fit" models previously used to describe the electrical conductivity of nanofluids.

## Introduction

Nanofluids are created by suspending nanometer size particles in a base fluid [1] and allow for the engineering of fluid properties by changing the type, size, and amount of particles. They have been proposed for advanced heat transfer applications such as fuel cell thermal management and power electronics cooling; however, many of these cooling applications require a low electrical conductivity fluid. While nanofluid thermal properties have received considerable attention both theoretically and experimentally [2], the interrelated and critical electrical properties have not.

The electrical conductivity of a suspension depends on the background electrolyte and particle size, charge, and volume fraction [3]. Nanofluids often utilize metal oxide nanoparticles such as ZnO, TiO_2_, or Al_2_O_3_. When dispersed in a fluid, these particles gain surface charge due to the protonation or deprotonation of a surface group such as a hydroxyl ligand (-OH) [4]. This surface charge, which can be adjusted in electrolyte solutions by altering the pH of the suspension [5,6] or chemically treating the particle surface [6], causes an electrical double layer (EDL) of counter-ions to form near the particle surface. For bulk suspensions that are salt-free, the only ions present are those from the charging process of the particles, which are counter-ions formed at the fluid-particle interface. For salt-free suspensions, the effective electrical conductivity is typically increased upon the suspension of particles since the ionic conductivity in the EDL is generally larger than that of the bulk solution [3].

Several researchers [7-11] have measured large increases in the electrical conductivity of nanofluids compared to the base fluid as the volume fraction [7-11] and temperature [10] are increased. For example, Ganguly et al. [10] reported a factor of 150 increase for 13-nm Al_2_O_3 _nanofluids at a volume fraction of 3%. This is a factor of 100 greater than the increase predicted by the Maxwell model [12]; since other models for the electrical conductivity of nanofluids do not currently exist, researchers have simply used a linear curve fit without physical interpretation [9,10].

A realistic model for nanofluid electrical conductivity must take into account nanoparticle size as well as the surface charge of the nanoparticle in suspension, neither of which is accounted for in the Maxwell model. Here, we apply electrokinetic models developed for colloidal suspensions to nanofluids for the first time to explain the large measured increases in electrical conductivity. Among many such models [13-17] we employ in particular the analytical model for spherical colloidal particles in a salt-free medium developed by Ohshima [16], to enable extraction of the physical parameters that govern nanofluid electrical conductivity. Using propylene glycol (PG) without any dispersants as a salt-free medium, we measure the electrical conductivities of 20, 40, and 60 nm diameter ZnO nanoparticle dispersions up to 7% volume fraction, applying Ohshima's model to determine the limiting ionic conductance of the system.

### Nanofluid preparation and characterization

#### Nanofluid preparation

In addition to serving as a salt-free medium, PG allows for higher nanoparticle volume fractions to be tested without dispersants than what is achievable in water-based nanofluids. PG-based nanofluids were prepared by Nanophase Technologies (Romeoville, IL, USA) at 7% volume fraction without any dispersants, and were diluted to 1, 3, and 5% for all particle sizes. By achieving volume fractions that are higher than any for which nanofluid electrical conductivity measurements have previously been reported, we are able to study effects such as counter-ion condensation that occur at high volume fractions and significantly impact electrical conductivity. The ZnO nanoparticles had a crystal phase of zincite (hexagonal) and an elongated morphology with an aspect ratio of approximately 3. The specific surface areas of the particles were 54, 33, and 18 m^2^/g for the 20-, 40-, and 60-nm particles, respectively. The suspensions were reported to be greater than 99% pure by the manufacturer.

#### Zeta potential measurement

The zeta potential of a suspension provides a measure of the electrokinetic potential of the EDL and strongly influences its electrostatic properties as described below. Nanofluid zeta potentials were measured using a Brookhaven Instruments Zeta Plus system (Holtsville, NY, USA) which correlates light scattering with average particle velocity in the presence of an applied electric field. Samples were maintained at 25°C, and the average zeta potential measured by five separate runs was recorded.

#### Electrical conductivity measurement

Nanofluid electrical conductivity was measured using a Model 72 handheld conductivity meter from Engineered Systems and Designs, Inc. (Newark, DE, USA). The meter included two insulated electrodes of fixed spacing and achieved a resolution of 0.1% and an accuracy of ±2.5% over the range of 0.2 to 20,000 μS/cm. The meter was calibrated using salt solutions with known electrical conductivities of 10, 74, 714, 2,000, 6,668, and 58,640 μS/cm. For the measurement of nanofluid samples, approximately 200 ml of the nanofluid was placed in a beaker which was submerged in a 25°C temperature bath. The electrodes were rinsed in tap water and then dipped into different beakers of distilled water to prevent any contamination between samples. The electrodes were then dipped into the sample beaker and stirred until the measured value stabilized. The rinsing process was repeated between each measurement, and the data for each sample was averaged over three measurements.

### Nanofluid electrical conductivity modeling

**Figure 1:**
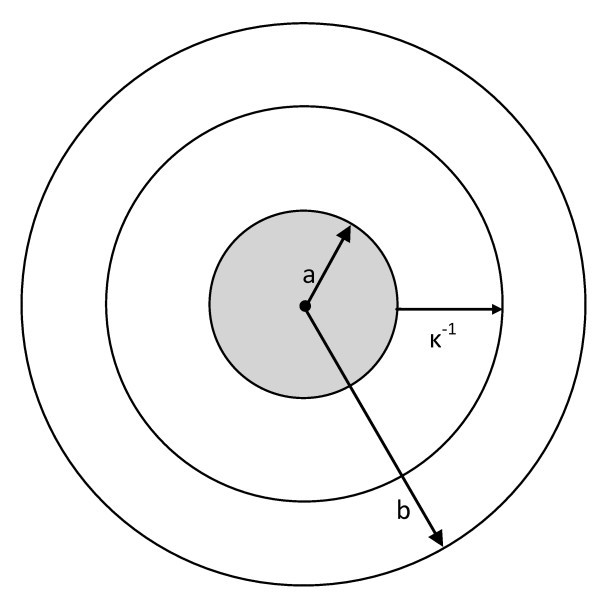
**Schematic of Kuwabara's cell model with particle radius, *a*, electrical double layer thickness, *κ***^**-1**^**, and surrounding shell of liquid medium with outer radius *b***.

Kuwabara's cell model [18], shown in Figure 1, is used to analyze the electrokinetic properties of colloidal suspensions. Each particle of radius, *a*, is surrounded by a virtual shell of the salt-free medium with of radius, *b*, such that the volume fraction, *ϕ*, equals (*a*/*b*)^3^. This model simplifies the electro-hydrodynamic interactions between the particles by representing an average particle and surrounding medium. This model does not account for overlapping EDLs and is commonly used to investigate the electrophoresis and sedimentation of colloidal suspensions of spherical particles. Based on this cell model, Ohshima [16] derived separate analytical expressions for the electrical conductivity *K *of a salt-free suspension that apply when the particle surface charge *Q *= 4*πε*_r_*ε*_0_*a*ζ is either less than or greater than a critical surface charge given by:(1)

where *ε*_r _is the relative permittivity of the medium, *ε*_0 _is the vacuum permittivity, ζ is the zeta potential, *z *is the valence of the counter-ion, *e *is the elementary electric charge, *k *is the Boltzmann constant, and *T *is the temperature in Kelvin. The surface charge, which exists at the interface between the medium and the particle, is not directly measured but rather is derived from the measured zeta potential. The condition given in (1) can then be expressed in terms of a critical zeta potential defined as:(2)

For Case 1 (ζ < ζ_crit_),(3)

and for Case 2 (ζ > ζ_crit_),(4)

where *λ *is the drag coefficient of the counter-ion [19]:(5)

for which *N*_*A *_is Avogadro's number and  is the limiting ionic conductance. As the volume fraction is increased, ζ_crit _is reduced. For Case 1, *K *is predicted to increase linearly with volume fraction since the addition of particles directly adds to the total charge. However, once the critical charge is reached and Case 2 becomes applicable, *K *is predicted to rise more slowly for increasing volume fraction due to counter-ion condensation effects that limit the effective particle charge to be less than the intrinsic charge [16]. Counter-ion condensation occurs due to an electrostatic coupling that induces an accumulation of counter-ions, which causes a renormalization of the charge at the surface. Therefore, increasing the amount of counter-ions in this case simply adds to the condensation region and leaves the charge and potential outside that region unchanged, causing the electrical conductivity to plateau.

## Results and discussion

### Zeta potential

The zeta potentials measured for the PG-based ZnO nanofluids are given in Table 1 and show only a slight dependence on particle size. Since the fluids were prepared using the same process, the zeta potential was controlled to be nearly constant. Using a counter-ion valence of 2 arising from the formation of Zn^2+ ^ions on the particle surface [20], the critical zeta potential can be calculated by Equation 2 to be 59.2 mV for 1% volume fraction, 45.0 mV for 3%, 38.5 mV for 5%, and 34.2 mV for 7%. The nanofluid samples for all particle sizes thus fall into Case 1 for 1% volume fraction and Case 2 for higher volume fractions.

**Table 1:** Measured zeta potential

2*a *(nm)	*ζ *(mV)
20	49.3 ± 0.1
40	48.6 ± 0.1
60	48.3 ± 0.1

### Derivation of electrokinetic parameters

The limiting ionic conductance () and counter-ion drag coefficient (*λ*) both relate to the counter-ion formed at the particle surface and are independent of the particle size and concentration. By assuming a relative permittivity for PG of 28.7 [21] and minimizing the root-mean-square error between the measured and the modeled electrical conductivities for all particle sizes and volume fractions (discussed below), we determine  and *λ *to be 54.4 S · cm^2^/mol and 6.18 × 10^-12 ^C^2^/(S · m^2^), respectively. These values are consistent with typical literature values for colloidal suspensions, which report  in the range of 40-350 S · cm^2^/mol [19].

### Comparison of experimental and predicted electrical conductivity

**Figure 2:**
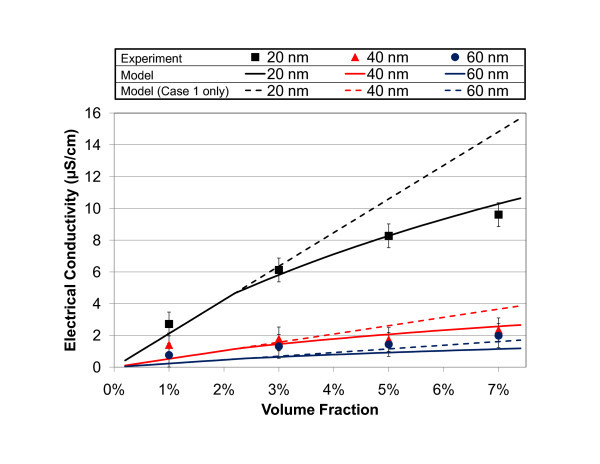
**Measured (solid symbols) and predicted (solid lines) electrical conductivity of propylene glycol-based ZnO nanofluids with 20-, 40-, and 60-nm diameter particles**. Predicted values are based on the colloidal salt-free suspension model given in Equations 3 and 4. A linear fit model (i.e., one which only assumes Case 1 and neglects counter-ion condensation effects) is also shown in dotted lines.

As shown in Figure 2, increasing the volume fraction by adding nanoparticles significantly increased the electrical conductivity with respect to that of the PG base fluid (*K *= 0.1 μS/cm). As predicted by Equations 3 and 4, smaller particles yielded a higher electrical conductivity at the same volume fraction. The electrical conductivity of the 20-nm particle suspension reached 9.60 μS/cm at 7% volume fraction, representing a nearly 100-fold increase over the base fluid.

Predicted electrical conductivities based on Equation 3 for 1% volume fraction and Equation 4 for higher volume fractions, using the measured zeta potentials given in Table 1, are also shown in Figure 2. Dotted lines are used to illustrate the error that occurs when extending Case 1 (e.g., linear increase with volume fraction) to higher volume fractions at which counter-ion condensation occurs. Condensation effects are particularly evident for the 20-nm nanoparticle samples, for which the measurement error is small compared to the change in electrical conductivity. Discrepancies between predicted and measured values may arise from the elongated particle geometry, which deviates from the spherical assumption of Ohshima's model, or from impurities in the suspension.

### Electrokinetic radius

The thickness of the EDL is strongly dependent on the ionic strength of the fluid medium and has a significant effect on electrical conductivity. This effect of ion concentration is captured by the electrokinetic radius (*κa*), which is the ratio of the particle radius (*a*) to the thickness of the EDL (*κ*^-1^) and is given by [16]:(6)

Equation 6 shows that *κa *is independent of particle size and increases with volume fraction. The electrokinetic radius calculated from the experimental data is shown in Table 2, and increases from 0.34 at 1% volume fraction to 0.92 at 7% volume fraction. While the electrokinetic radius (ratio of the EDL thickness to the particle radius) does not change with particle radius, the EDL thickness does. Table 3 shows the calculated EDL thicknesses (*κ*^-1^) for nanoparticles of different sizes. The thickness decreases with increasing volume fraction and decreasing particle size. The minimum thickness of the EDL is calculated to be 10.9 nm for a 20-nm particle at 1% volume fraction, and the maximum thickness is calculated to be 88.9 nm for a 60-nm particle at 1% volume fraction. For all nanofluid volume fractions studied here, *κa *was found to be less than 1, implying the presence of a relatively thick EDL compared to that of suspensions in high ionic strength electrolytes. For double layers in this size range, it has previously been noted [3] that models for electrical conductivity can have relatively higher error due to the complexities of the increased surface conductance near the particle which create a non-linearity with volume fraction, potentially leading (along with the geometric and impurity uncertainties mentioned above) to the slight discrepancies between predicted and measured electrical conductivities observed.

**Table 2:** Calculated electrokinetic radius

*Φ *(%)	*κa*
1	0.34
3	0.59
5	0.77
7	0.92

**Table 3:** Calculated thickness (nm) of the EDL (*κ*^-^^1^) for different volume fractions and particle diameters (2*a*)

*Φ *(%)	2*a *(nm)		
	20	40	60
1	29.6	59.3	88.9
3	16.9	33.9	50.8
5	13.0	26.0	38.9
7%	10.9	21.7	32.6

## Conclusions

Electrical characterization of nanofluids with volume fractions as high as 7% demonstrated electrical conductivities that fall significantly below simple linear fit models that have previously been applied. The experimental data showed an increase in electrical conductivity with increasing volume fraction and with decreasing particle size at constant volume fraction. The leveling off of electrical conductivity observed at high volume fractions is consistent with counter-ion condensation effects that are captured by a model previously developed for colloidal suspensions in salt-free media. Optimizing such counter-ion condensation effects in nanofluids could potentially increase their applicability in technologies for which low electrical conductivity is required.

## Abbreviations

EDL: electrical double layer; PG: propylene glycol.

## Competing interests

The authors declare that they have no competing interests.

## Authors' contributions

SW carried out the electrical conductivity studies and modeling, and drafted the manuscript. AS and KP participated in the design of the study and its coordination. All authors read and approved the final manuscript.
